# Streamlined identification of strain engineering targets for bioprocess improvement using metabolic pathway enrichment analysis

**DOI:** 10.1038/s41598-023-39661-x

**Published:** 2023-08-10

**Authors:** Joan Cortada-Garcia, Rónán Daly, S. Alison Arnold, Karl Burgess

**Affiliations:** 1 Institute of Quantitative Biology, Biochemistry and Biotechnology, School of Biological Sciences, University of Edinburgh, Edinburgh, EH8 9AB UK; 2 Institute of Infection, Immunity and Inflammation, Glasgow Polyomics, University of Glasgow, Glasgow, G61 1QH UK; 3 Ingenza Ltd., Roslin Innovation Centre, Roslin, EH25 9RG UK

**Keywords:** Biological techniques, Biotechnology, Computational biology and bioinformatics

## Abstract

Metabolomics is a powerful tool for the identification of genetic targets for bioprocess optimisation. However, in most cases, only the biosynthetic pathway directed to product formation is analysed, limiting the identification of these targets. Some studies have used untargeted metabolomics, allowing a more unbiased approach, but data interpretation using multivariate analysis is usually not straightforward and requires time and effort. Here we show, for the first time, the application of metabolic pathway enrichment analysis using untargeted and targeted metabolomics data to identify genetic targets for bioprocess improvement in a more streamlined way. The analysis of an *Escherichia coli* succinate production bioprocess with this methodology revealed three significantly modulated pathways during the product formation phase: the pentose phosphate pathway, pantothenate and CoA biosynthesis and ascorbate and aldarate metabolism. From these, the two former pathways are consistent with previous efforts to improve succinate production in *Escherichia coli*. Furthermore, to the best of our knowledge, ascorbate and aldarate metabolism is a newly identified target that has so far never been explored for improving succinate production in this microorganism. This methodology therefore represents a powerful tool for the streamlined identification of strain engineering targets that can accelerate bioprocess optimisation.

## Introduction

Strain engineering and media optimisation have proven to be a successful tool for bioprocess improvement, allowing commercial feasibility of several biotechnological products. Two of the most well-known examples are penicillin—going from laboratory production soon after its discovery in 1928^1^ to large-scale production in 1943 with 100-to-1000-fold increase in product titres (up to 1.8 g L^−1^)^2^—and artemisinic acid—precursor of the antimalarial drug artemisinin, for which heterologous expression was demonstrated in *Saccharomyces cerevisiae* up to 100 mg L^−1^ and was further improved to 25 g L^−1^ titres^3,4^.

With the advent of “-omics” technologies, system-wide analysis of biological samples is possible, increasing the potential for bioprocess improvement with better guided strain and media optimisation. The uses and advantages of each -omics discipline have been reviewed elsewhere^5–7^. In this work, the focus is placed on metabolomics, the global analysis of the metabolome^8^, which provides detailed information on the small molecules in a biological system, and therefore has the most relevance for small molecule bioprocess improvement. Some examples of different ways in which metabolomics has been used to enhance bioprocesses are given below.

Nitta et al. used unsupervised (principal component analysis) and supervised (orthogonal partial least squares regression) multivariate analysis on 74 targeted metabolites to find gene targets for the improvement of 1-butanol production in *Escherichia coli* (*E. coli*)^9,10^. In the first of the two studies, acetyl-CoA was identified as a bottleneck, which was resolved with the overexpression and optimisation of the RBS region of the *atoB* gene, which converts acetyl-CoA into acetoacetyl-CoA, resulting in a significant improve in 1-butanol titres. In the second study, glyoxylate was found to accumulate. The knockout of *aceA*, the first gene in the glyoxylate shunt, resulted in a 39% increase in 1-butanol titres in the cultivation conditions tested. In a different study, Kawaguchi et al. used targeted metabolomics to find intracellular bottlenecks in the co-consumption of glucose and l-arabinose in *Corynebacterium glutamicum* ATCC 31831^11^. The authors analysed 138 metabolites of central metabolism, with their conclusions based on 29 of these metabolites. Their analysis led to the overexpression of *pyk* and deletion of the *araR* repressor, achieving the simultaneous consumption of both substrates at the same rate.

Other authors looked at a smaller set of targeted metabolites for strain optimisation. For example, George et al. used targeted metabolomics to look at eight compounds of the isoprenoid pathway in *E. coli* to improve the heterologous production of three C5 alcohols with potential use as biofuels, particularly by engineering the Shine-Dalgarno sequence of the *nudB* gene, significantly alleviating the bottleneck of isopentenyl diphosphate^12^. Barton et al. performed a targeted ^13^C labelling analysis using LC–MS to look at the intracellular levels of two compounds of the 1,4-butanediol (BDO) formation pathway and five up-stream by-products^13^. This way, the authors found the last two steps to be the bottleneck of BDO formation. Subsequent genetic engineering of these two steps led to improved BDO formation.

Some studies have also looked at the availability of different target nutrients during the bioprocess in order to identify and prevent specific limitations to improve process performance. For example, Korneli et al. looked at 22 metabolites—19 of which were amino acids—and identified amino acid limitation during the production of GFP in *Bacillus megaterium*^14^, and Ho et al. looked at the composition of six fatty acids—and at least 11 other intracellular metabolites—produced by the marine microalga *Chlamydomonas* sp. JSC4 under different conditions of salinity and nitrogen starvation, allowing an increase in lipid accumulation by adjusting the cultivation conditions^15^.

In the examples mentioned above—and others^16–19^—metabolomics was used as a targeted tool to look at specific metabolites and pathways, in most cases using unit mass resolution mass spectrometers—such as single- and triple-quadrupoles—thus limiting the potential of finding engineering targets for bioprocess improvement to those based on prior knowledge of the biological system. However, untargeted metabolomics offers the possibility to find targets for bioprocess optimisation in a wider metabolic context in a more unbiased fashion, particularly when used with high-resolution accurate mass (HRAM) mass spectrometry. For example, Xu et al. performed an untargeted metabolomics analysis to compare a *pho13Δ* mutant of *S. cerevisiae*—with a higher capacity to catabolise xylose—with its parental strain. A total of 134 intracellular metabolites were identified, and sedoheptulose-7P—a metabolite from the pentose phosphate pathway (PPP) outside carbohydrate metabolism—showed the most significant difference between both strains. Mutants overexpressing the PPP gene *TAL1* at different degrees were constructed, and their *TAL1* expression levels were positively correlated to their respective xylose consumption rates^20^.

Another study used metabolite profiling to determine which nutrients were limiting in a bioprocess for recombinant IgG4 antibody production using Chinese hamster ovary (CHO) cells, a discovery experiment better suited to untargeted metabolomics due to the unknown composition of the medium^21^, and Xia et al. used untargeted and targeted metabolomics to compare the production by *Streptomyces tsukubaensis* of FK506—a polyketide used as immunosuppressant—in a high and low productivity media^22^. Multivariate analysis of the metabolomics results with partial least squares (PLS) allowed the authors to identify and supplement limiting nutrients in the medium, increasing FK506 production. The 13 nutrients most significantly correlated with FK506 biosynthesis included coenzyme A (CoA) esters, shikimate, amino acids, pyruvate, lactate and PPP intermediates. Once again, these would have been difficult to identify by targeting the product biosynthetic pathway.

As the examples above show, untargeted metabolomics offers the possibility to identify key metabolites for bioprocess improvement that are outside the product biosynthetic pathway, which are commonly missed when targeted methods are used focusing on prior biological knowledge about the system. However, untargeted metabolomics produces very large datasets from which it is challenging to prioritise metabolic reactions and pathways for modification. A useful tool for dealing with and obtaining useful information from untargeted metabolomics data is pathway enrichment analysis, which analyses groups of compounds that work together to carry out a biological process—like a metabolic pathway^23,24^, and ranks them in terms of their statistical significance and importance. Although pathway enrichment analysis has historically been applied more frequently to genomics, transcriptomics and proteomics data^25–27^, it has also been applied in the field of metabolomics. However, most of these publications are clinical studies^28–32^.

There have been some recent studies where metabolic pathway enrichment analysis (MPEA) has been used with metabolomics data for bioprocess improvements. For example, Morris et al. compared four different fed-batch cultivation conditions resulting in differences in monoclonal antibody titres in a CHO bioprocess^33^. The authors then manually looked in the KEGG database^34^ at the pathways of the 10 most significant metabolites, as identified by PLS-discriminant analysis (PLS-DA), to find titre inhibitors and promoters that might be modulated by changing the bioprocess feeding strategy. With this approach, however, constraining pathway analysis to the 10 most significant metabolites significantly limits the vast analytical power of metabolomics. In another example, Alden et al. used untargeted metabolomics to find metabolites that accumulated in the culture medium of CHO fed-batch processes. Using MPEA with a modified Fisher’s exact test, the authors found three pathways that were enriched in the cell line with the lowest cell density profile of their study, including aminoacyl-tRNA biosynthesis, tryptophan and histidine metabolism. Further investigation into 11 putatively annotated metabolites that accumulated in the culture medium led the researchers to hypothesise that products of tryptophan metabolism could behave as inhibitors of cell growth. The authors finished by suggesting targeting the tryptophan pathway with genetic engineering, or lowering the concentration of tryptophan in the cultivation medium^35^. These examples show how MPEA can be used for bioprocess improvement, however, this is still a line of research that is underexplored, particularly for the identification and prioritising of genetic targets for bioprocess optimisation.

MPEA can be performed in different ways, similar to the examples described above for targeted and untargeted metabolomics. Mainly, the analysis can focus on comparing a case and a control group, such as high- and low-productivity conditions or strains^9,10,15,20,22^, or focus on the dynamic changes of different metabolites throughout the course of the fermentation^11,12,14,21^. Although not mutually exclusive, the first type of analysis can be more tailored to identifying media deficiencies, performance biomarkers and genetic differences between differently performing strains, whereas the second type can be used to improve an established working fermentation process further by, for example, identifying the accumulation of by-products, the presence of inhibitors, the (in)activation of specific metabolic pathways or the depletion of substrates.

In this study, a commercial *E. coli* succinate production process was analysed with a combined targeted and untargeted metabolomics method using HRAM mass spectrometry, and the results were used to perform MPEA throughout the time course of the fermentation to identify potential targets for bioprocess optimisation in an unbiased fashion.

## Results

### Escherichia* coli* succinate fermentation process

**Figure 1:**
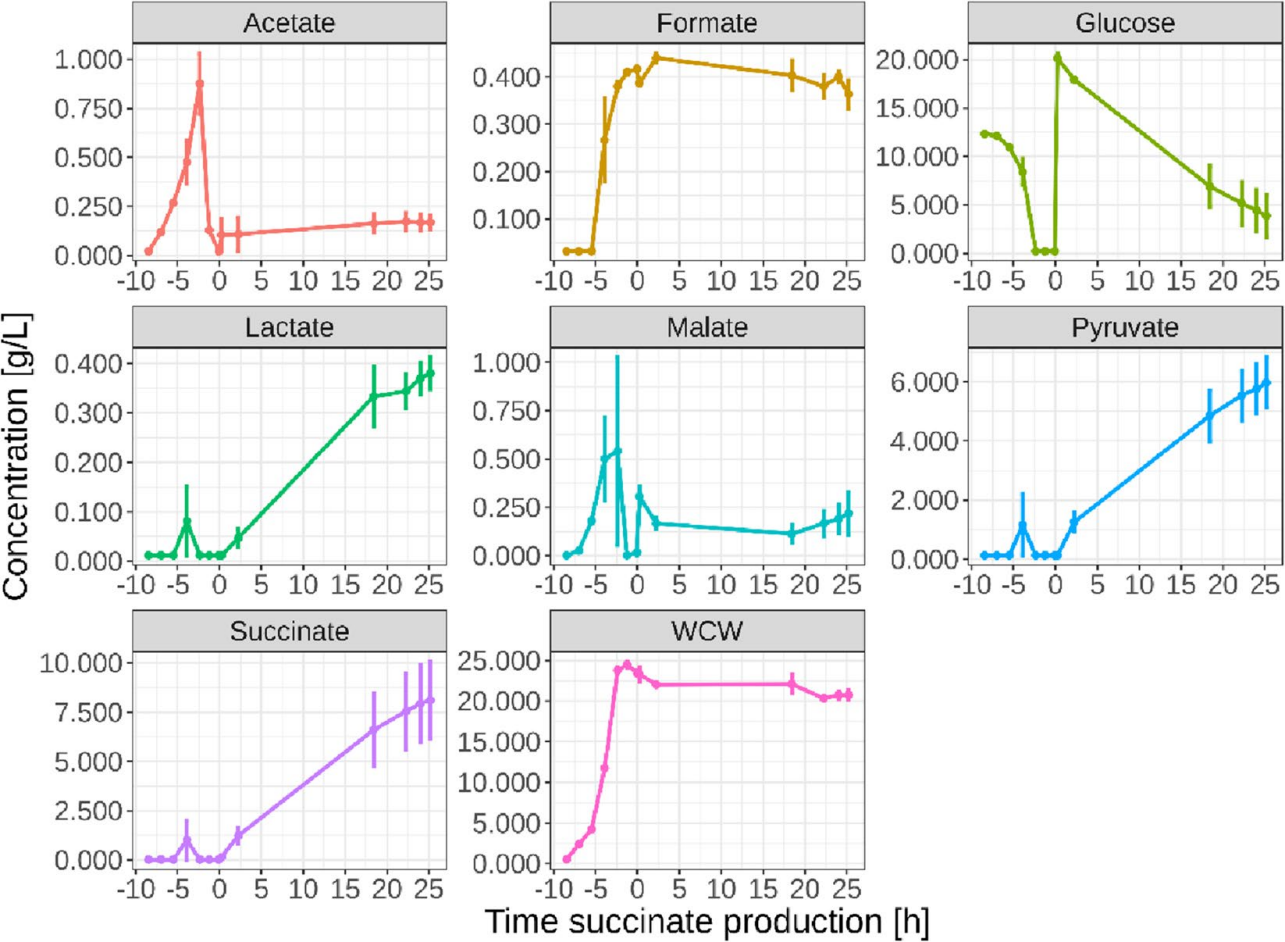
Profile of the *E. coli* succinate fermentation process showing the main parameters measured by HPLC–UV/Vis-RI. Time is indicated with respect to the beginning of the succinate production phase. Error bars represent the standard error of the mean of the three replicates (n = 3).

Three *E. coli* dual-phase succinate fermentation replicates were performed and samples were taken throughout the course of the fermentation for metabolomics analysis with LC–MS. Furthermore, the extracellular concentration of glucose and the main fermentative products was also determined by HPLC–UV/Vis-RI analysis (Fig. 1). The fermentation process is split into two distinctive parts. The first phase is an aerobic batch process, where all the glucose from the medium is consumed for biomass growth, with little production of fermentative products. Under aerobic conditions with glucose excess, acetate is formed in *E. coli* due to overflow metabolism, and part of this acetate is excreted out of the cell to prevent osmotic stress due to the accumulation of negative charges in the cytoplasm. After glucose depletion, this acetate can be reimported inside the cell and be consumed as a source of carbon and energy^36–38^. A small amount of lactate, malate, pyruvate and succinate was measured in one of the three replicates in the middle of the biomass exponential growth phase, potentially due to a temporary limited supply of oxygen. However, the concentration of these organic acids went back down before the end of the biomass growth phase. The second phase of the bioprocess is an anaerobic production phase (biotransformation), where additional glucose is added to the medium and this is converted into succinate and other fermentative products, being pyruvate the main by-product. The *E. coli* strain used in this process cannot sustain cell growth on glucose under anaerobic conditions, which can be appreciated by the plateau and slight decrease in the biomass wet cell weight (WCW) measurements during the anaerobic phase of the process.

### LC–MS analysis of fermentation samples

The three fermentation experiments were analysed by LC–MS to try to identify targets for improving succinate production. A total of 13 samples from each replicate fermentation were analysed using both intracellular and extracellular fractions of the samples (see Supplementary file “Metabolomics data.xlsx”). The Principal Component Analysis (PCA) plot of the samples for both intra- and extracellular analysis shows good clustering of the different time points of the three fermentation replicates, demonstrating reproducible data (Fig. S1 in the Supplementary materials), despite the lower succinate production for one of the three fermentation runs. The combined untargeted and targeted analysis resulted in a total of 6341 annotated and 92 identified metabolites (Table S1 in the Supplementary materials and see also the Supplementary file “Metabolomics data.xlsx”). Metabolite identification was performed by matching the retention time and accurate mass to reference standards following the Metabolomics Standards Initiative (MSI) classification system^39^.

### Metabolic pathway enrichment analysis

The metabolomics results were used to perform pathway enrichment analysis in order to find potential genetic targets for bioprocess optimisation without bias from preconceived process-specific biological knowledge. MPEA was performed using the Pathway Activity Level Scoring (PALS) application in the Polyomics integrated Metabolomics Pipeline (PiMP) online platform^40^ (date of use: 08 Jul 2020). PALS ranks significantly changing metabolite groups over different sample sets using the pathway level analysis of gene expression for metabolomics (mPLAGE) algorithm, which converts m/z features into formulae and matches them to pathways^41^ and the KEGG library^34^ (see Supplementary file “Pathway enrichment analysis data.xlsx”). The output of the mPLAGE algorithm is a p-value that has taken into account the multiple comparisons over all the pathways in the KEGG library. The different time points of the fermentation data were grouped by triplicate measurements and used for pathway enrichment analysis. For every triplicate time point of the fermentation, the algorithm evaluates pathways that are significantly different when compared to the first triplicate time point. This way, every metabolic pathway assessed is assigned a significance level (p-value) for every fermentation sample (except for the first time point). Each p-value indicates the probability that there is no difference between the time points for the metabolites on the pathway. The first sample analysed is a time point at the early stages of the aerobic phase of the fermentation (right after inoculation for extracellular samples and 3 h after inoculation for intracellular samples. See the “Methods” section for more details). By doing a time-course comparison to an early time point in the fermentation where the cells had abundant access to glucose and oxygen, it is possible to identify changes in metabolic regulation at the different stages of the fermentation (e.g. end of biomass growth, beginning or end of succinate production phase, etc.). This information can be used to put the results in the context of the experiment and aid in the identification of engineering targets.

**Figure 2:**
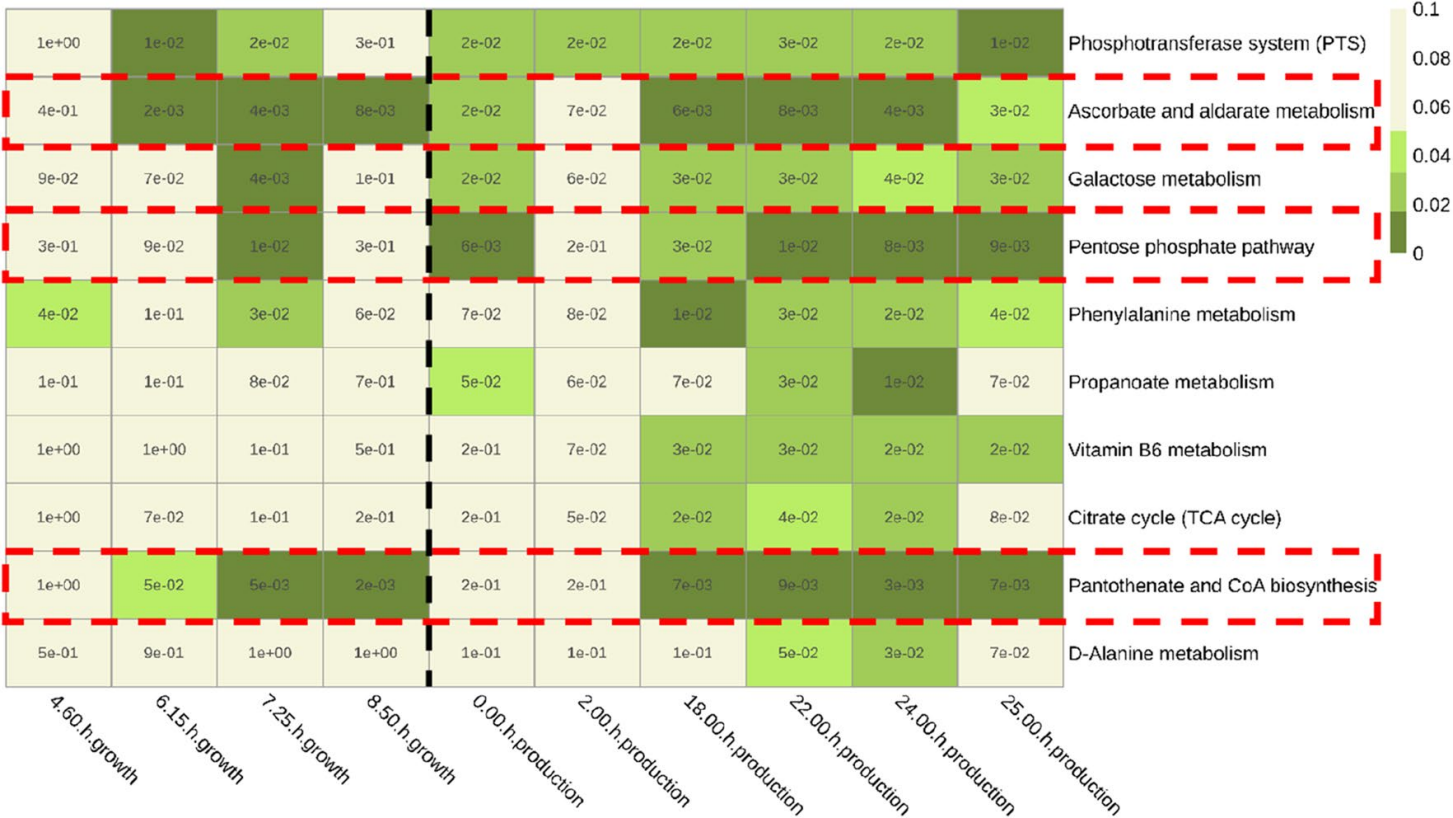
Top 10 pathways with the lowest mean p-value in the succinate production phase based on MPEA using the mPLAGE algorithm for intracellular metabolites found with a combined targeted and untargeted metabolomics method. Pathway names are shown on the y-axis (in increasing average p-value in the production phase going down) and time points of the fermentation process on the x-axis. The values of the cells are the p-values (n = 3) comparing each time point to the first time point of the fermentation analysed (3 h after the beginning of the aerobic batch phase). The resulting colour corresponds to the level of significance: green (p-values ≤ 0.05) and beige (p-values > 0.05). The vertical black dashed line indicates the transition from the aerobic growth phase to the anaerobic succinate production phase. The horizontal red dashed lines indicate the three pathways highlighted as potential targets for strain engineering.

To find genetic targets for improved succinate production, the mean p-value of each pathway (as compared to the first time point) was calculated across all time points in the succinate production phase. This mean p-value was used as ranking criteria (rather than focusing on extremely small p-values at individual time points) in order to find pathways with consistent statistical significance throughout the production phase and avoid the impact of potential outliers. Intracellular and extracellular metabolite fractions were analysed separately. The ten pathways with the highest level of significance (lowest mean p-value) resulting from analysing intracellular metabolites are represented as a heatmap in Fig. 2. These include several pathways from amino acid, carbohydrate, nucleotide and vitamin metabolism.

More significant changes were observed in the extracellular fraction samples (Fig. S2 in the Supplementary materials). This is caused by a significant increase in the number of metabolites found in the extracellular fraction as the fermentation progresses—potentially due to cell lysis, particularly under oxygen limitation or absence, because the *E. coli* strain used cannot sustain biomass growth on glucose anaerobically. The large number of significant pathways makes the selection of specific ones with significant changes in metabolic levels based on the extracellular fraction more challenging. For this reason, the selection of potential genetic targets was done purely based on the MPEA performed with the intracellular metabolites.

### Selection of potential genetic targets

Pathways showing high metabolic changes during the succinate production phase could be potential targets for metabolic reshuffling. Either due to the removal of by-products or the elimination of metabolic bottlenecks, the modulation of these pathways could potentially lead to higher titres or yields. From the 10 pathways in Fig. 2, three pathways were identified to have particularly low p-values during the succinate production phase: the pentose phosphate pathway, ascorbate and aldarate metabolism and pantothenate and CoA biosynthesis (marked with red boxes in Fig. 2). The criteria to select these three particularly was that they all had at least three time points in the succinate production phase with a p-value ≤ 0.01.

**Figure 3:**
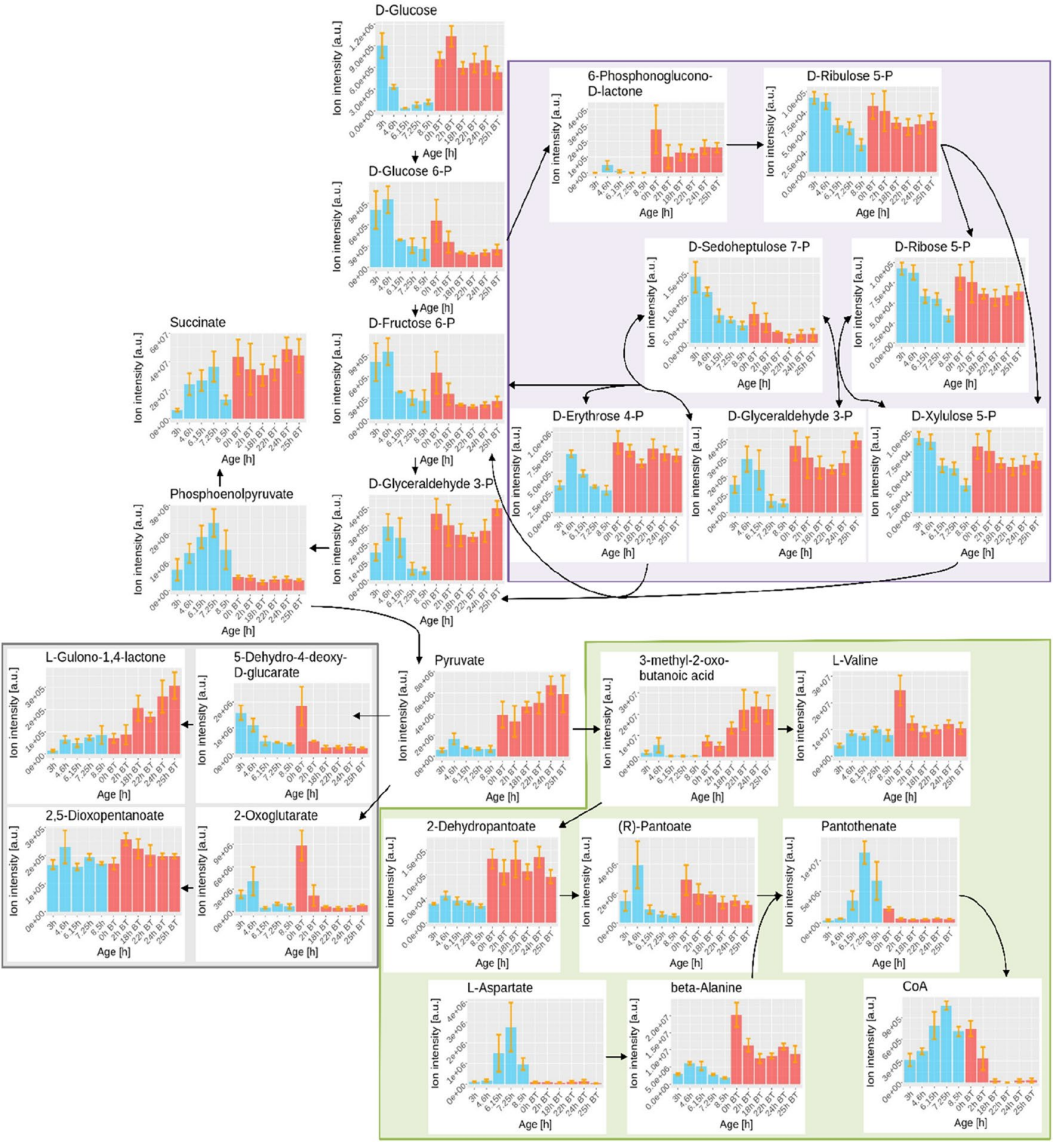
Dynamic evolution of intracellular metabolites of the three pathways identified as potential targets for genetic engineering: the pentose phosphate pathway (purple), pantothenate and CoA biosynthesis (green) and ascorbate and aldarate metabolism (grey). A simplified version of the pathways is shown for ease of reference. The results show both phases of the bioprocess: the aerobic bacterial growth phase (blue) and the succinate production phase (red). Orange error bars represent the standard error of the mean (n = 3).

The metabolomics results for these pathways are shown in Fig. 3. Several metabolites from the PPP show a sudden increase in intracellular levels at the beginning of the succinate production phase and in many cases the levels remain high, with the exception of sedoheptulose-7P. The sudden increase at the beginning of the production phase is also observed for the precursors of pantothenate. However, pantothenate itself shows a decrease in intracellular intensity at the transition from the aerobic growth phase to the anaerobic production phase. Similarly, CoA levels drop significantly between 2 and 18 h of succinate production, indicating that CoA availability can be a potential bottleneck for succinate production, as suggested by Lin et al.^42^. Finally, 5-dehydro-4-deoxy-d-glucarate and 2-oxoglutarate—both involved in ascorbate and aldarate metabolism—show a sudden increase in intracellular levels at the beginning of the production phase, followed by a sudden decrease. However, l-gulono-1,4-lactone shows a progressive increase in intracellular intensity throughout the succinate production phase, indicating intracellular accumulation. Trying to minimise this accumulation can, therefore, be another potential genetic target to improve succinate production.

### PLS-DA for identifying targets of succinate improvement

**Figure 4:**
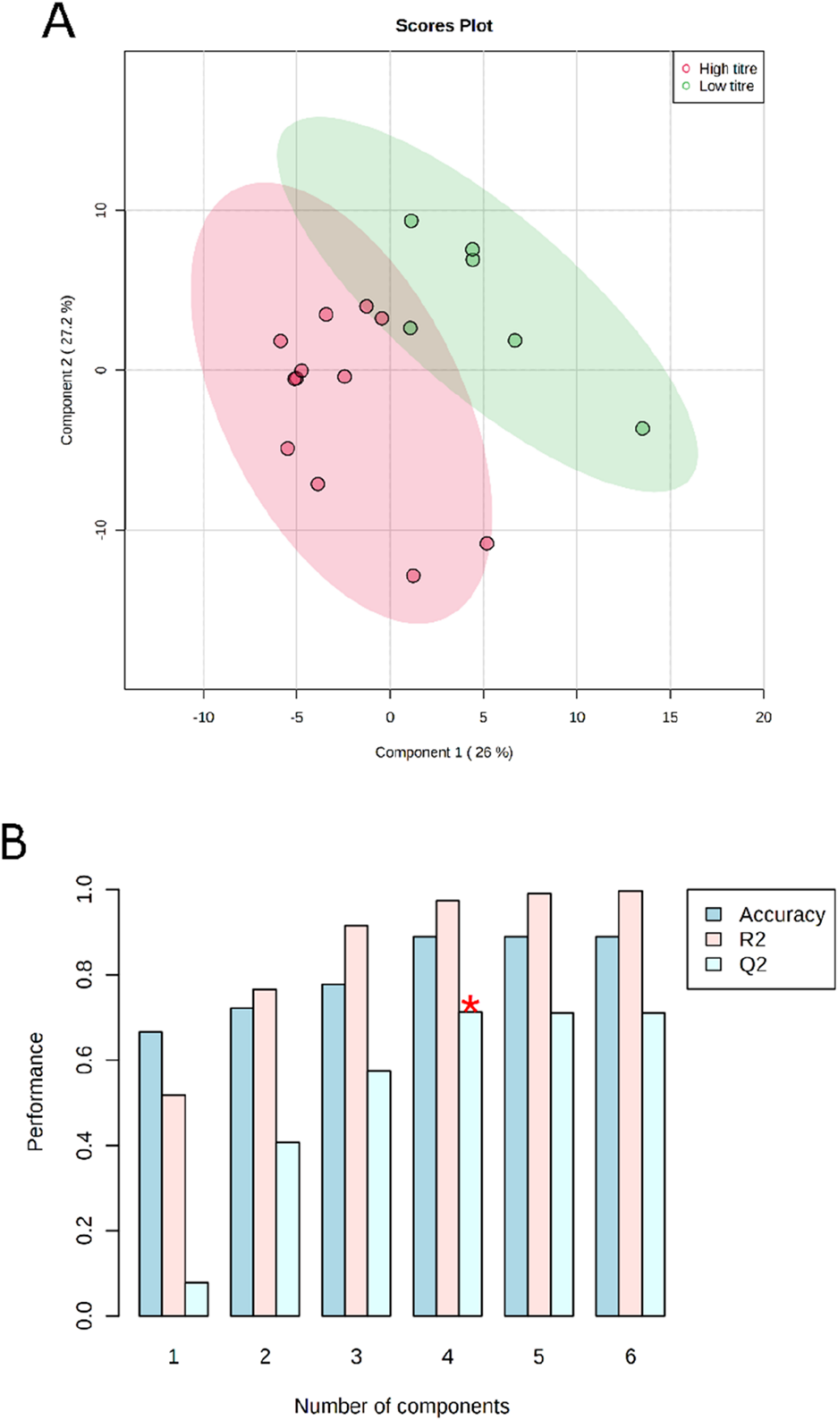
(**A**) Scores plot between the latent variables (components) 1 and 2 for the PLS-DA of the three fermentation replicates analysed, which were split into two experiments with a high succinate titre (n = 12) and one with low succinate titre (n = 6). The explained variances of each latent variable are shown in brackets. (**B**) PLS-DA classification using different number of latent variables (components). The red star indicates the best classifier based on maximising Q^2^.

Other authors have used PLS regression to identify targets for bioprocess engineering^9,22^. Therefore, PLS-DA was chosen as a benchmark analytical method to compare to the findings of the metabolic pathway enrichment analysis. For this, the three fermentation replicates described in this work were split into two groups, the first group containing the two experiments with higher succinate titres and the second group containing the experiment with a lower titre (10.05 ± 0.64 SD and 4.23 g/L final succinate concentration, respectively). Then, the samples from the succinate production phase of the bioprocess were analysed by PLS-DA to identify which metabolites contribute the most to the differences in succinate titre (Fig. 4). The samples from the aerobic growth phase were not included in the analysis to avoid discrimination based on the large metabolic differences between the aerobic biomass growth phase and the anaerobic succinate production phase. Succinate was also removed from the data set before performing PLS-DA in order to prevent biasing group discrimination. Good separation of the two sample groups was observed (Fig. 4, top), and the four first latent variables were selected to build the model, based on maximising the cross-validated coefficient of determination (Q^2^ = 0.7123, R^2^ 0.9738, Fig. 4, bottom).

**Figure 5:**
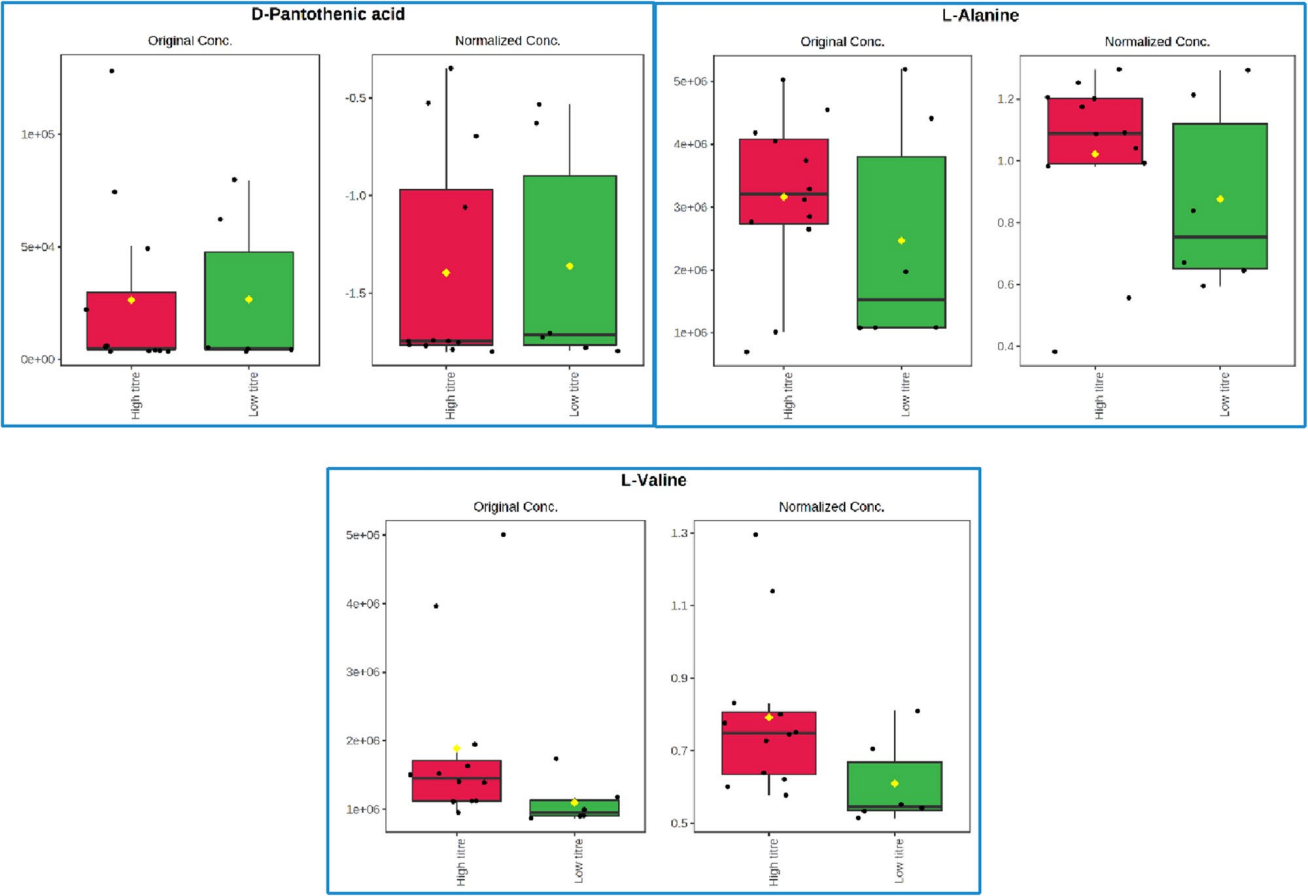
Three metabolites involved in pantothenate and CoA biosynthesis found important for discrimination of a successful *E. coli* succinate fermentation run according to the PLS-DA model (VIP score > 1 in at least one of the four latent variables) and with a high level of annotation confidence. The boxplots show the peak intensity of the three metabolites in the two sample groups: high (red) and low (green) succinate titre (n = 12 and 6, respectively). The peak intensity values are shown as black dots, the black horizontal line represents the population median and the yellow diamond represents the population mean. For each metabolite, the subplot on the left shows the results with the original peak intensity data, and the subplot on the right the results after log_10_ transformation and normalisation of the data.

From a total of 3989 mass spectrometry signals analysed in PLS-DA, 495 were found significant with a variable importance in projection (VIP) score > 1^43^ in the first latent variable (Supplementary file PLSDA summary.xlsx). The VIP score is a common measure in PLS-DA of the relative importance of each metabolite with respect to the total variation of the latent variable. From these significant signals, 270 had unknown annotations and many of the 225 remaining had multiple possible metabolic annotations. Those metabolites with the highest level of annotation confidence and a VIP score above 1 in the four first latent variables were investigated. Metabolites with a high level of annotation confidence were defined as either having retention time and accurate mass matched to a reference standard, or accurate mass and fragmentation pattern matched to metabolomics library data. The 38 metabolites that met the criteria above are gathered in Table S2 (see Supplementary file PLSDA summary.xlsx), and most of them belong to amino acid metabolism (18 out of the 38 metabolites) and nucleotide metabolism (10 out of 38), which is a much more reduced breadth of metabolic pathways than the findings from Fig. 2. Nevertheless, coinciding with the results from MPEA, three metabolites involved in pantothenate and CoA biosynthesis were found important for discrimination of a successful *E. coli* succinate fermentation run according to the PLS-DA model, namely d-pantothenic acid, l-alanine and l-valine (Fig. 5), strengthening the hypothesis that this pathway could be a potential target of interest for bioprocess optimisation. While l-alanine and l-valine show clearer differences between both sample groups (higher levels for the higher succinate titre group), the levels of d-pantothenic acid are more similar in both groups, which is also reflected in the latter having a lower VIP score in the first two latent variables (see Supplementary file PLSDA summary.xlsx). However, it is worth mentioning that PLS-DA looks at individual metabolites and does not reveal any biological information of the relationship between them, whereas enrichment analysis groups features (metabolites in this case) under common biological themes, making it more robust to reveal real biological differences between sample groups^44^. That is, for analysing the PLS-DA results, the three abovementioned metabolites were manually linked to the same metabolic pathway assisted by the findings from MPEA from Fig. 3. In other words, MPEA makes it easier to put metabolomics results in a biological context.

## Discussion

Metabolomics is an expanding field that has been previously applied to identify potential targets for genetic engineering to improve bioprocesses^9–13,20^. However, in most cases, this is still performed with targeted metabolomics using unit mass resolution single- or triple-quadrupole instruments looking at pre-selected intermediates from the biosynthetic pathway of the product of interest, thus limiting the vast detection capacity of mass spectrometry. Some examples are available using untargeted metabolomics^20–22^, however, these tend to identify potential genetic targets using multivariate statistical tools—often PCA or PLS—applied directly to the metabolomics data without using information from metabolic pathways. As this study shows, without the context of biological pathways it can be very hard to interpret and extract useful information from untargeted metabolomics data using multivariate statistical analysis. This was demonstrated by developing a PLS-DA model to discriminate between higher and lower succinate titre runs in an *E. coli* bioprocess, based on targeted and untargeted intracellular metabolomics data. Overall, although the PLS-DA model was able to achieve good separation of the two sample groups, the large number of significant signals and the high level of uncertainty in metabolite annotation makes it difficult to identify genetic targets for succinate production improvement. Focusing on those metabolites with a higher confidence in annotation it was possible to identify metabolites from amino acid, nucleotide and pantothenate and CoA metabolism as being important for group discrimination. However, this is a time- and effort-intensive exercise that left behind 448 of the 495 (90.51%) significant features according to the PLS-DA model.

Conversely, the use of MPEA allows to exploit metabolomics data further. By considering correlated changes in different compounds of specific pathways, certain enrichment analysis tools such as PALS can be used to analyse targeted and untargeted metabolomics to identify significantly modulated pathways during the bioprocess in a more streamlined way. Importantly, as MPEA highlights sets of metabolites that are consistently regulated, it is more robust to individually misannotated metabolites than techniques that look at individual metabolites without considering any biological context (such as PLS-DA). Furthermore, the PALS tool used in this work uses formulae rather than identifications to safeguard even more against the impact of potential misannotations.

The *E. coli* succinate production bioprocess was analysed with MPEA and the results pointed at three different pathways with significantly different levels of metabolites during the bioprocess: the pentose phosphate pathway, pantothenate and CoA biosynthesis and ascorbate and aldarate metabolism.

Interestingly, the PPP has previously been reported to play an important role during succinate production in *E. coli.* For example, Lu et al.^45^ calculated the carbon flux down the PPP during succinate production using LC–MS and HPLC measurements and using a stoichiometric model in an *E. coli* AFP111 dual phase bioprocess using 13C-labelled glucose as a carbon source. Using a higher percentage of CO_2_ during the succinate production phase increased both succinate production and the percentage of carbon flux channelled towards the PPP. In another study, Zhu et al. found that increased activity of the enzyme transketolase in the PPP led to an increase in succinate titre and yield in *E. coli* ATCC 8739 derived strains. The succinate titre and yield improved even further increasing the activity of the enzyme transhydrogenase, which can convert NADPH generated from the PPP into NADH—which can then be used for succinate production^46^. The same research group performed a further study where the authors generated a library of PPP genes under the regulation of a constitutive M1-93 promoter and different ribosome binding sites to generate different *E. coli* ATCC 8739 derived variants with different expression levels of the PPP genes. Their results indicate that increasing the expression levels of PPP genes can lead to higher succinate yields and titres, but that a fine tuning of the expression levels gives the best results^47^. These examples are in agreement with the identification, in this work, of the PPP as a potential target for succinate improvement using MPEA, validating this methodology for the identification of pathways for bioprocess improvement.

There is less prior literature about manipulating the pantothenate and CoA biosynthetic pathways to increase succinate production. However, Lin et al. showed increased succinate production in *E. coli* GJT001 derived strains by overexpressing the enzyme pantothenate kinase, which catalyses the first step in the conversion of pantothenate to CoA^42^. Succinate production was increased even further with the co-overexpression of either phosphoenolpyruvate carboxylase or pyruvate carboxylase, which catalyse the conversion of phosphoenolpyruvate and pyruvate into oxaloacetate. The authors attributed the increase in succinate production to a higher intracellular availability of acetyl-CoA and CoA. In all experiments, the fermentation medium was supplemented with 5 mM pantothenate, therefore, genetic engineering to increase the levels of intracellular pantothenate would also be required on top of the changes indicated by the authors to increase succinate production. This study suggests that genetic manipulation of the pantothenate and CoA pathway can, indeed, lead to an increase in succinate production.

Finally, as far as ascorbate and aldarate metabolism is concerned, no examples were found in the literature showing the manipulation of this pathway in *E. coli* for increased succinate production. Therefore, this study might be the first to identify this pathway as a potential target for genetic engineering to achieve improved succinate production in *E. coli*.

This work is the first example of the application of MPEA using both targeted and untargeted metabolomics to identify potential strain engineering targets for bioprocess improvement. The examples above show that the targets identified with this methodology are relevant and coincide with previous successful attempts to improve succinate production in *E. coli.* The three pathways identified in this work are outside the succinate biosynthetic pathway, showing how untargeted metabolomics can identify important pathways for product formation, even if these are initially not known to have an impact on the bioprocess. Untargeted metabolomics has the potential to accelerate bioprocess optimisation, and pathway enrichment analysis is a useful tool to help analyse and interpret the results obtained with this technology putting them in a biological context.

Looking forward, it is important to mention that, although MPEA is a powerful tool for the identification of significantly regulated pathways, it does not indicate how these need to be modified to achieve the desired strain phenotype. Indeed, biological regulation is complex, with many factors affecting metabolic and protein levels. Therefore, predicting and modulating phenotypes is still difficult even when knowing which metabolic pathways are highly regulated in a bioprocess. Nevertheless, MPEA can be a powerful tool to streamline and speed up the Design-Build-Test-Learn cycle of strain engineering, i.e. the identification of engineering targets.

## Methods

### Bacterial strain

All experiments described in this article were carried out using a proprietary industrial *E. coli* strain (Ingenza Ltd., UK) previously described^48^, based on the *E. coli* NZN111 strain with deletions of the pyruvate-formate lyase (*pflB*) and lactate dehydrogenase (*ldhA*) genes^49^.

### Growth media

All 5 L scale fermentation experiments were carried out with a batch phase for biomass formation using a defined minimal medium containing 11.90 g/L glucose as the sole carbon source, 2.00 mM MgSO_4_, a mix of salts solution (2.00 g/L (NH_4_)_2_SO_4_, 14.60 g/L K_2_HPO_4_, 3.60 g/L NaH_2_PO_4_·2H_2_O, 0.50 g/L (NH_4_)_2_H-citrate), a mix of trace elements (1.0 mg/L CaCl_2_·2H_2_O, 20.06 mg/L FeCl_3_, 0.36 mg/L ZnSO_4_·7H_2_O, 0.32 mg/L CuSO_4_·5H_2_O, 0.30 mg/L MnSO_4_·H_2_O, 0.36 mg/L CoCl_2_·6H_2_O, 44.60 mg/L Na_2_EDTA·2H_2_O), antibiotics (100 mg/L kanamycin, 34 mg/L chloramphenicol) and antifoam (33.33 µL/L polypropylene glycol P-2000). Shake flask overnight cultures were prepared using the same medium but with 10.00 g/L glucose and no antifoam.

### Fermentation process conditions

All fermentation experiments were carried out in a 5 L Applikon stirred tank fermenter (ADI 1030 Bio Controller, 1035 Bio Console), and the process consisted of an initial aerobic batch phase where the minimal medium was primarily used for biomass formation, followed by a 24 h anaerobic succinate production phase as previously described^48^.

#### Inoculum

Fermentation inocula were prepared by inoculating 50 µL of cell bank into 100 mL of growth medium in a 500 mL baffled shake flask and incubated at 37 °C and 165 rpm for 17–17.5 h.

#### Aerobic batch phase for biomass growth

The fermentation was started by inoculating 100 mL of overnight culture into 3 L of growth medium in the 5 L fermenter for a starting OD_600_ of 0.21 ± 0.025. During biomass growth the conditions were maintained at 37 °C temperature, 500–900 rpm agitation (controlled to keep the dissolved oxygen (DO) > 30%), 4.00 L/min air (1.33 vvm) and pH 7.0 ± 0.1, controlled with 2.00 M H_2_SO_4_ and 28% w/v NH_4_OH.

#### Anaerobic succinate production phase

At the beginning of the production phase, glucose from a 500 g/L solution and sodium bicarbonate from a 100 g/L solution were added to the fermenter as a single bolus addition to a final concentration of 20 g/L and 5 g/L respectively in the vessel, as previously described^50^. The sodium bicarbonate provides soluble CO_2_, which is required for the conversion of PEP to oxaloacetate^51^. Once the glucose and sodium bicarbonate were added to the fermenter, the sparged air was replaced by pure (99.8%) CO_2_ at 0.50 L/min (0.17 vvm), agitation was set to 300 rpm, temperature at 37 °C and pH at 7.0 ± 0.1 controlled with 2.00 M H_2_SO_4_ and 28% w/v NH_4_OH.

### Biomass measurement

Biomass levels were reported as OD_600_ and wet cell weight (WCW). The former was the measured optical density at 600 nm wavelength. The latter was determined by spinning down 1 mL of sample for 5 min at 14,462 g twice in a pre-weighed Eppendorf tube, removing the supernatant and weighing the resulting pellet. The weight of the pellet in g/L was calculated from gravimetric difference.

### Metabolites extraction for LC–MS analysis

Samples for off-line liquid chromatography-mass spectrometry (LC–MS) analysis were removed from the bioreactor and collected into dry-ice-cold universal vials, placed briefly on an ethanol dry ice bath for a fast cold sample quenching and immediately spun down twice at 4 °C and 13,000 g for 10 min. The supernatant and cell pellet were collected as extracellular and intracellular fractions respectively and stored at − 80 °C until further extraction for LC–MS analysis.

#### Extracellular fractions

Extracellular fraction extractions were prepared by diluting 10 µL of sample into 400 µL of 1:3:1 chloroform:methanol:water (C:M:W). The samples were then mixed vigorously in a chilled microtube mixer for 5 min and then centrifuged for 3 min at 13,000*g* and 4 °C. At this point, 360 µL of supernatant were transferred into a new microtube and stored at − 80 °C until LC–MS analysis. 25 µL of supernatant of each extracted sample were combined into one single vial to generate a pooled sample. During handling, the 1:3:1 C:M:W extraction solvent and the samples were kept on an ethanol dry ice bath.

#### Intracellular fractions

Prior to extraction, intracellular fractions were washed by resuspending the cell pellets in 1 mL of sterile phosphate buffer solution. The phosphate buffer was removed by spinning down the samples twice for 10 min at 13,000 g and 4 °C. For metabolite extraction, 200 µL of 1:3:1 C:M:W were added for every 5 mg of WCW pellet, thus normalising all samples by their biomass concentration. Cell pellets were resuspended by pipetting, and then the samples were mixed vigorously in a chilled microtube mixer for 1 h, before being centrifuged for 3 min at 13,000*g* and 4 °C. At this point, 200 µL of supernatant were transferred into a new microtube. The samples were further diluted by adding 200 µL 1:3:1 C:M:W and stored at − 80 °C until LC–MS analysis. 25 µL of supernatant of each extracted sample were combined into one single vial to generate a pooled sample. During handling, the 1:3:1 C:M:W extraction solvent and the samples were kept on an ethanol dry ice bath. The first two fermentation samples had a very small biomass pellet, which made the extraction process impractical, particularly the resuspension of the pellet in the amount of extraction solvent required for sample normalisation. For this reason, these first two time points from the intracellular fraction were not included in the analysis.

### LC–MS method

Metabolite separation was performed using a zwitterionic hydrophilic interaction liquid chromatography (ZIC^®^-pHILIC) column (Merck SeQuant^®^) (150 mm × 4.6 mm, 5 µm particle size) equipped with the corresponding guard column (20 mm × 2.1 mm, 5 µm particle size) (Merck SeQuant^®^). A linear gradient was applied to the column, running from 80 to 20% solvent B over 15 min, followed by a 2 min wash with 5% solvent B, and 9 min re-equilibration with 80% solvent B, where solvent B was acetonitrile and solvent A (the remaining percentage) was 20 mM ammonium carbonate in water. The total flow rate was 300 µL/min, column temperature was maintained at 25 °C, sample injection volume was 10 µL, samples were maintained at 4 °C for the duration of the analysis and a HESI probe was used on the ion source.

Metabolite detection was done in a high-resolution Thermo Scientific™ Q Exactive™ Orbitrap mass spectrometer at 70,000 resolution, mass range 70–1050 m/z in polarity switching mode with a spray voltage of ± 3.8 kV. Capillary temperature was set to 320 °C, sheath gas 40 a.u., AGC target 1 × 10^6^ a.u. and the lock masses in positive and negative mode were 144.9822 m/z and 100.9856 m/z, respectively.

Fragmentation was performed on pooled samples by isolating ions in a 1.2 m/z window and fragmentation with stepped HCD collision energy of 24.8, 60.0 and 94.8% for both polarities with 17,500 resolution and AGC target 1 × 10^5^ a.u. Top 10 ions (intensity threshold 1.3 × 10^5^) were selected for fragmentation and then added to a dynamic exclusion window for 15 s.

### Metabolomics data processing and analysis

Raw mass spectrometry files were converted into .mzXML files in profile mode with the open-source software ProteoWizard^52^ (Version 3.0). Further data processing and analysis was performed using the PiMP online platform^40^ (date of use: 08 Jul 2020). Peak detection and filtering were set to 3 ppm of the theoretical monoisotopic mass, minimum intensity to 5000, noise to 0.8, retention window to 0.05 and minimum number of detections to 3. Peak retention time was corrected using the Obiwarp algorithm^53^ from the xcms package (Version 1.48.0).

#### Metabolite annotation and identification

Metabolite annotation was performed using a local copy of the KEGG database^34^ (Metabolomics Standards Initiative (MSI) class 2/3 annotation^39^), the fragmentation library (MSI class 2 annotation) and by matching the retention time and accurate mass to reference standards, which are listed in the Supplementary file Std_list_Metabolites.xlsx.

### HPLC–UV/Vis-RI analysis

HPLC coupled to UV/Vis and refractive index detectors (HPLC–UV/Vis-RI) analysis was carried out using a Rezex™ ROA Organic Acid H + ion-exclusion column (Phenomenex^®^) (300 mm × 7.8 mm) equipped with a Carbo-H4 guard column (SecurityGuard™) (3.0 mm i.d.). An isocratic method was applied to the column, running a 5 mM H_2_SO_4_ mobile phase solution for 30 min. The total flow rate was 800 µL/min, column temperature was maintained at 65 °C, sample injection volume was 10 µL and samples were maintained at 4 °C for the duration of the analysis. The HPLC–UV/Vis-RI data was extracted as a .csv file and was further analysed using the ggplot2 package^54^ (Version 3.3.3) in the statistical software environment R (Version 3.6.1). Metabolite levels below the limit of quantification were replaced by 1/5 of the minimum metabolite concentration quantified.

### Metabolic pathway enrichment analysis

MPEA was performed using the PALS application in PiMP (date of use: 08 Jul 2020) using the mPLAGE algorithm and a local copy of the KEGG database^34^. Pathways with p-values of 0.05 or below were considered statistically significant across experimental groups compared. The experimental groups compared were the different time points, and each pairwise comparison was done comparing any given time point with the first sample of the fermentation. In order to reduce the dimensionality of the analysis, for each pathway, the p-values of all the samples in the succinate production phase were averaged together and this mean p-value was used to find the 10 “most significant” pathways (lowest mean p-value). These 10 “most significant” selected pathways were displayed as a heatmap showing the p-value of each pathway for each sample. Heatmaps were created using the gplots package^55^ (Version 3.1.3) in the statistical software environment R (Version 4.0.4). MPEA for intracellular and extracellular samples was performed separately.

### PLS-DA

PLS-DA was performed in MetaboAnalyst (date of use: 08 Sep 2022) with R (Version 4.1.3). Constant features across all samples were deleted and missing values were replaced by 1/5 of the minimum positive values of their corresponding variables. Variables with near-constant values throughout the experiment conditions were filtered using the interquartile range, and the data was log_10_ transformed and normalised by the median to adjust for systematic differences among samples (Figs. S3 and S4 in the Supplementary materials). The number of latent variables for the PLS-DA model was chosen by maximising Q^2^ using tenfold cross validation. Those features with a VIP score above 1 in the first latent variable were considered as significant for group discrimination when analysing the results.

### Supplementary Information

Supplementary Information 1.

Supplementary Information 2.

Supplementary Information 3.

Supplementary Information 4.

Supplementary Information 5.

## Data Availability

The data that support the findings of this study are available in the Supplementary materials and in MetaboLights at http://www.ebi.ac.uk/metabolights/MTBLS6667, reference number MTBLS6667.
