# Supplementary material for: Streamlined identification of strain engineering targets for bioprocess improvement using metabolic pathway enrichment analysis
Source: Sci Rep. 2023 Aug 10;13:12990. doi: 10.1038/s41598-023-39661-x (PMC10415327; doi:10.1038/s41598-023-39661-x)
Supplement: Supplementary file 5 — Supplementary Information 5. [file 41598_2023_39661_MOESM5_ESM.docx]

Supporting Information

Streamlined identification of strain engineering targets for bioprocess improvement using metabolic pathway enrichment analysis

Joan Cortada-Garcia ^1^, Rónán Daly ^2^, S. Alison Arnold ^3^ and Karl Burgess* ^1^

1 Institute of Quantitative Biology, Biochemistry and Biotechnology, School of Biological Sciences, University of Edinburgh, Edinburgh EH8 9AB, United Kingdom
2 Institute of Infection, Immunity and Inflammation, Glasgow Polyomics, University of Glasgow, Glasgow G61 1QH, United Kingdom
3 Ingenza Ltd., Roslin Innovation Centre, Roslin EH25 9RG, United Kingdom

Table of contents

SUPPLEMENTARY FIGURES S2

[SUPPLEMENTARY TABLES S6](#_Toc591638716)

SUPPLEMENTARY FIGURES


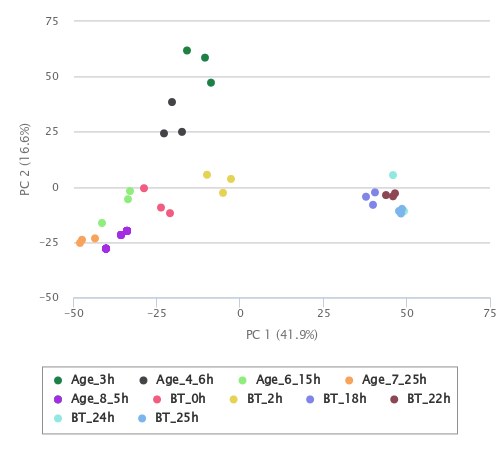

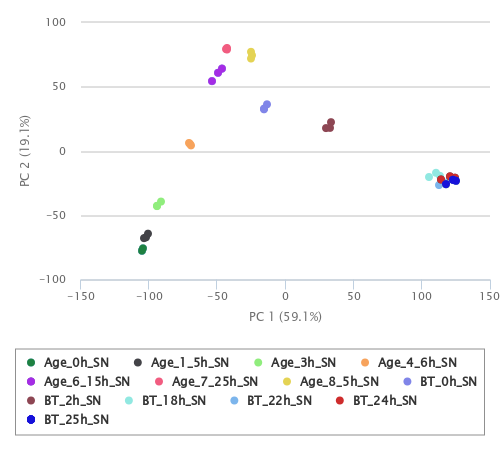


Figure S 1. PCA plot of triplicate E. coli fermentation samples for both intracellular (**left**) and extracellular (**right**) fractions. Note that the first two time points for the intracellular fraction were not analysed because the low amount of biomass in the sample made the metabolite extraction impractical. Age denotes the fermentation time during the biomass formation phase, and BT denotes the succinate production phase. Abbreviations: BT: biotransformation; SN: supernatant


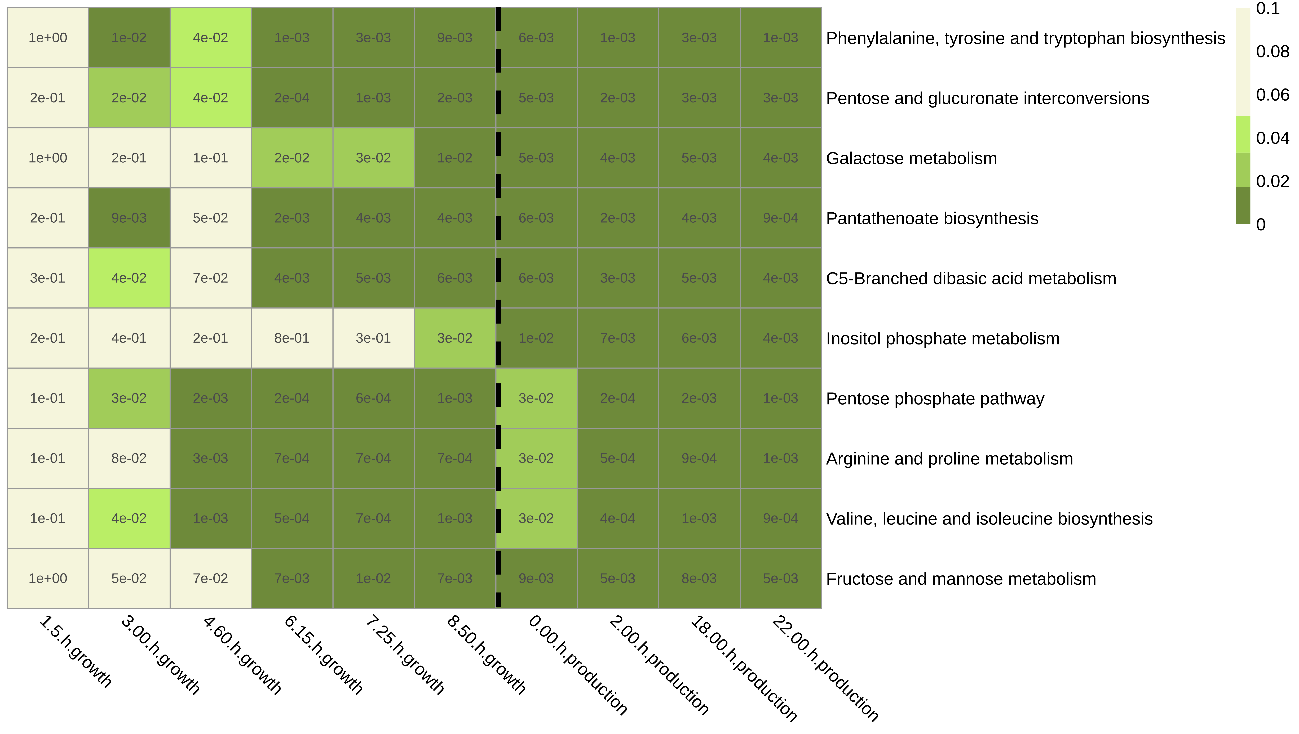


Figure S 2. Top 10 pathways with the lowest mean p-value in the succinate production phase based on metabolic pathway enrichment analysis using the mPLAGE algorithm for extracellular metabolites found with a combined targeted and untargeted metabolomics method. Pathway names are shown on the y-axis (in increasing average p-value going down) and time points of the fermentation process on the x-axis. The values of the cells are the p-values (n=3) comparing each time point to the first time point of the fermentation. The resulting colour corresponds to the level of significance: green (p-values ≤ 0.05) and beige (p-values > 0.05). The vertical black dashed line indicates the transition from the aerobic growth phase to the anaerobic succinate production phase.


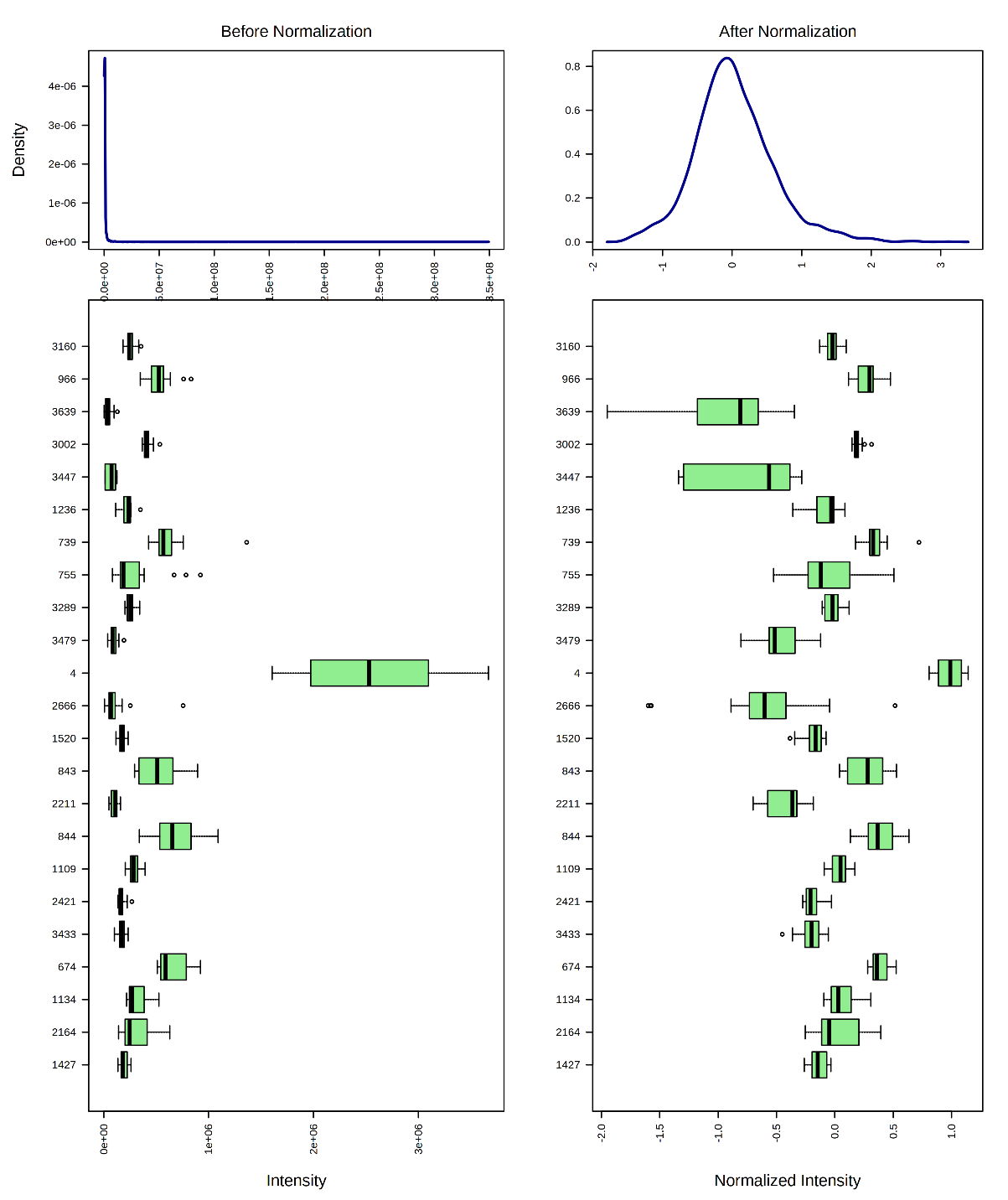


*Figure S 3. Box plots and kernel density plots before and after normalisation. The boxplots are plotted by mass spectrometry feature, including all the samples analysed. At most, 50 features are displayed due to space limit, and these are labelled according to the peak id number (see Supplementary file PLSDA summary.xlsx). The density plots are based on all samples. Selected methods: row-wise normalisation to sample median; data transformation: Log_10_ transformation; Data scaling: N/A.*


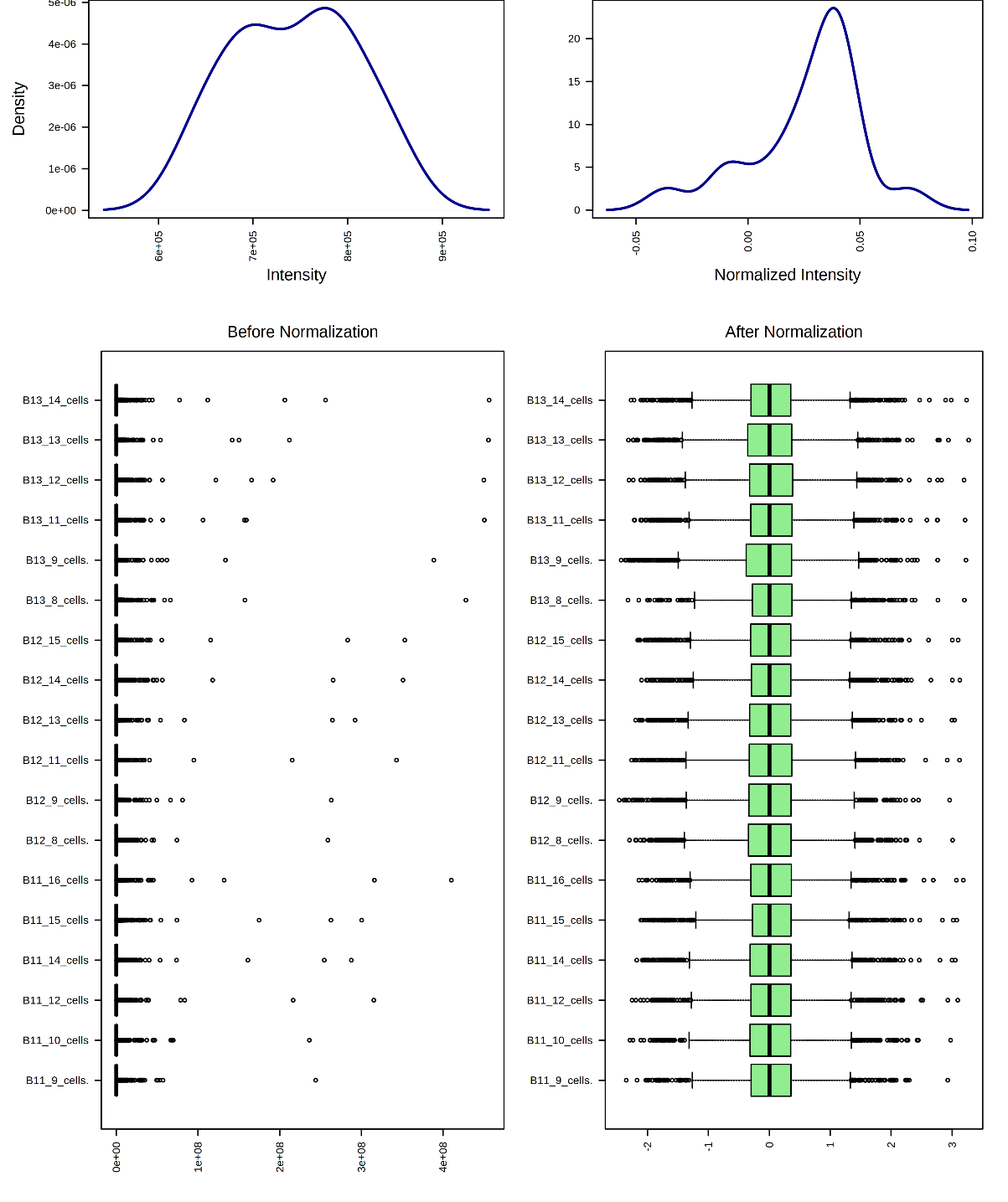


*Figure S 4. Box plots and kernel density plots before and after normalisation. The boxplots are plotted by sample, including all the mass spectrometry features analysed. Samples are labelled with the fermentation name (B11, B12 or B13) and sample number followed by the indicator “cells”, denoting the intracellular fraction. The density plots are based on all samples. Selected methods: row-wise normalisation to sample median; data transformation: Log_10_ transformation; Data scaling: N/A.*

SUPPLEMENTARY TABLES

Table S 1. Summary of the LC-MS results of the triplicate E. coli succinate production fermentation process analysed on PiMP. Annotated compounds are based on accurate mass and/or fragmentation data. Identified compounds are matched against reference standards. The mathematical union of both intra- and extracellular fractions contains all elements in either fraction without repetitions. To avoid repetitions, m/z values were rounded to 4 decimal points and retention times to the closest 10^th^ second.

| Parameter | Intracellular fraction | Extracellular fraction | Mathematical union of intra- and extracellular fractions |
| --- | --- | --- | --- |
| Peaks detected | 3549 | 4538 | 7334 |
| Total compounds | 4453 | 4872 | 6433 |
| Annotated compounds | 4372 | 4801 | 6341 |
| Identified compounds | 81 | 71 | 92 |
